# Central nervous system complications in SARS-CoV-2-infected patients

**DOI:** 10.1007/s00415-023-11912-x

**Published:** 2023-08-13

**Authors:** Zhonggui Li, Danyu Lin, Xiaoshuang Xu, Xiaohuan Liu, Jieli Zhang, Kaixun Huang, Feiyifan Wang, Jianfeng Liu, Zhi Zhang, Enxiang Tao

**Affiliations:** Department of Neurology, The Eighth Affiliated Hospital, Sun Yat-Sen University (Futian, Shenzhen), Shenzhen, China

**Keywords:** COVID-19, CNS complications, Encephalitis, Demyelination, ADEM, MS, MOG

## Abstract

**Objective:**

To investigate the clinical manifestations, treatment and prognosis of COVID-19-associated central nervous system (CNS) complications.

**Methods:**

In this single-centre observation study, we recruited patients with COVID-19-associated CNS complications at the neurology inpatient department of the Eighth Affiliated Hospital, Sun Yat-Sen University (Futian, Shenzhen) from Dec 2022 to Feb 2023. Patients were analysed for demographics, clinical manifestations, cerebrospinal fluid properties, electroencephalographic features, neuroimaging characteristics, and treatment outcome. All patients were followed-up at 1 and 2 months after discharge until Apr 2023.

**Results:**

Of the 12 patients with COVID-19-associated CNS complications, the CNS symptoms occur between 0 days and 4 weeks after SARS-CoV-2 infection. The most common CNS symptoms were memory deficits (4/12, 33%), Unresponsiveness (4/12, 33%), mental and behavioural disorders (4/12, 33%). Seven of 12 cases can be categorized as probable SARS-CoV-2 encephalitis, and 5 cases can be described as brainstem encephalitis, acute disseminated encephalomyelitis, optic neuritis, multiple sclerosis or tremor probably associated with SARS-CoV-2 infection. Six patients received antiviral therapy, and 11 patients received glucocorticoid therapy, of which 3 patients received human immunoglobulin synchronously. Nine patients recovered well, two patients had residual neurological dysfunction, and one patient passed away from complications associated with tumor.

**Conclusion:**

In this observational study, we found that the inflammatory or immune-related complications were relatively common manifestations of COVID-19-associated CNS complications, including different phenotypes of encephalitis and CNS inflammatory demyelinating diseases. Most patients recovered well, but a few patients had significant neurological dysfunctions remaining.

## Introduction

Beginning in December 2019 [1], a novel coronavirus swept through the world rapidly, taking a major toll on the global economy and healthcare industry. This novel coronavirus was named SARS-CoV-2, and the disease caused by it was named COVID-19; this disease was declared to be a global pandemic by the World Health Organization (WHO) on March 11, 2020 [2, 3]. As of 26 March 2023, over 761 million confirmed cases and over 6.8 million deaths have been reported globally [4]. Although SARS-CoV-2 mainly affects the respiratory tract and is characterized by respiratory symptoms such as fever, dry cough and difficulty breathing, there is increasing evidence that SARS-CoV-2 infection can cause severe neurological symptoms and complications [5–8]. While central nervous system complications of COVID-19 are relative rare, the total number of patients may grow very large as the epidemic progresses.

The reported CNS complications related to COVID-19 mainly include encephalitis [9–11], acute myelitis [12, 13], acute disseminated encephalomyelitis (ADEM) [14], cerebrovascular disease [15, 16], seizure [7, 17], and tremors, among others [18, 19]. At present, the available information on CNS complications related to COVID-19 is mostly from case reports. Special management of highly infectious diseases faces many challenges, such as isolation treatment, resulting in clinical data deficiencies of many early case reports, such as lack of cerebrospinal fluid (CSF) analysis, neuroimaging, and follow-up. In this study, we summarized and analysed the clinical data of 12 COVID-19 patients with CNS complications, including clinical manifestations, CSF properties, electroencephalograms (EEGs), neuroimaging characteristics, and treatment outcomes, paving the way to a much more detail clinical understanding of the CNS complications associated with COVID-19.

## Methods

### Study design and participants

For this single-centre observation study, we recruited patients with COVID-19-associated CNS complications from all COVID-19 patients admitted to the neurology inpatient department of the Eighth Affiliated Hospital, Sun Yat-Sen University (Futian, Shenzhen) during the Omicron pandemic wave from Dec 2022 to Feb 2023. The inclusion criteria were as follows. First, the patients met the WHO COVID-19 case definitions or the diagnostic criteria for novel coronavirus infection in the “Novel Coronavirus Infection Diagnosis and Treatment Protocol (Trial Version 10)” issued by the National Health Commission of the People's Republic of China in 2023. Second, the patients had CNS complications such as meningitis, encephalitis (including encephalopathy), ADEM, or myelitis. While cerebrovascular diseases were not included in this study. Finally, the CNS complications occurred within 6 weeks post-COVID-19 infection. The study complied with the Declaration of Helsinki and was approved by the Ethics Committee of the Eighth Affiliated Hospital, Sun Yat-Sen University. All patients signed written informed consent respectively.

### Research methods

All patients enrolled in our study underwent detailed neurological examination. Demographic information, clinical manifestation, blood and CSF laboratory results, EEG abnormalities, MRI changes, treatment plans, and outcomes were collected from the electronic medical records and management system of the Eighth Affiliated Hospital, Sun Yat-Sen University. Clinical outcomes were followed up to Apr 2023. All data were examined by two experienced neurological doctors.

### Statistical analysis

Clinical information was summarized through descriptive statistics. Continuous variables are presented as the median (range). Count variables are presented as numbers (percentages). Sector graphs and timeline charts were drawn by Microsoft Excel (version 2302 Build 16.0.16130.20332). Statistical analysis was performed with IBM SPSS Statistics (version 26.0).

## Results

### Demographic characteristics

A total of 12 patients were diagnosed with COVID-19-associated CNS complications, including 7 males and 5 females. The median age of these patients was 46.5 years (range 15–87). CNS symptoms occur between 0 days and 4 weeks after SARS-CoV-2 infection. Seven (58.3%) patients had medical comorbidities, including cardiovascular disease such as hypertension (2/12) or atrial fibrillation (1/12); endocrine system disease such as diabetes (2/12) or hyperthyroidism (1/12); malignant tumour (1/12); and nervous system disease (3/12) such as cerebral infarction, cognitive impairment or meningitis (Table 1).

**Table 1:** The characteristics of 12 patients with CNS complications during or following COVID-19 infection

Variables	Patients with CNS complications
Age, median (range)	46.5 (15–87)
Male, *n*/*N* (%)	7/12 (58.3%)
Neurological manifestations, *n*/*N* (%)	
Headache	1/12 (8.3%)
Dizziness	2/12 (16.7%)
Gustatory and olfactory dysfunctions	1/12 (8.3%)
Memory deficits	4/12 (33.3%)
Unresponsiveness	4/12 (33.3%)
Mental and behavioral disorders	4/12 (33.3%)
Disorders of consciousness	3/12 (25%)
Visual field defect	1/12 (8.3%)
Aphasia	1/12 (8.3%)
Insomnia	3/12 (25%)
Tremor	1/12 (8.3%)
Sensory or movement disorders	3/12 (25%)
Ataxia	1/12 (8.3%)
Epilepsy	2/12 (16.7%)
Dysarthria	1/12 (8.3%)
Medical comorbidities, *n*/*N* (%)	
History of any neurological disorder	3/12 (25%)
Malignant tumor	1/12 (8.3%)
Hypertension	2/12 (16.7%)
Diabetes mellitus	2/12 (16.7%)
Hyperthyroidism	1/12 (8.3%)
Use of antiviral drugs, *n*/*N* (%)	7/12 (58.3%)
Use of glucocorticoid, *n*/*N* (%)	11/12 (91.7%)
Use of immunoglobulin, *n*/*N* (%)	3/12 (25%)

### Clinical features

**Fig. 1:**
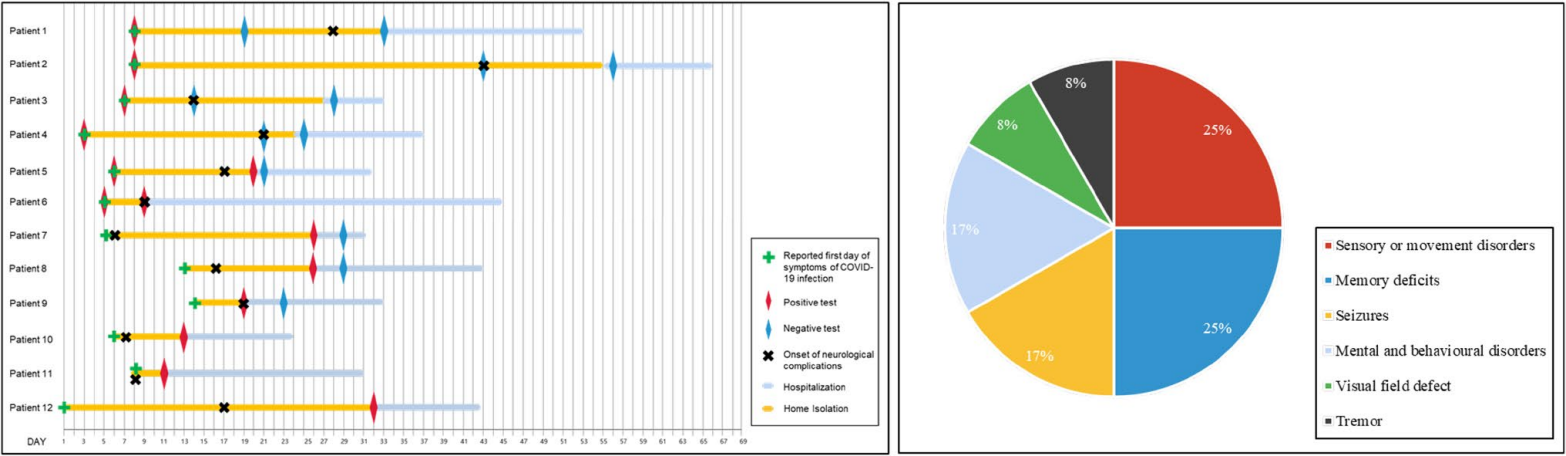
Left panel: timeline of onset of COVID-19 infection, neurological complications, hospitalization, home isolation and confirmation tests of SARS-CoV-2 among the 12 patients. Right panel: initial neurological symptoms of the 12 patients

The onset of neurological complications occurred 0–28 days after SARS-CoV-2 infection (Fig. 1). The most common CNS symptoms were memory deficits (4/12, 33%), unresponsiveness (4/12, 33%), and mental and behavioural disorders (4/12, 33%), followed by sensory or movement disorders (3/12, 25%), disorders of consciousness (3/12, 25%) and insomnia (3/12, 25%) (Table 1). Sensory or movement disorders (3/12, 25%) and memory deficits (3/12, 25%) were the most common initial neurological symptoms, followed by seizures (2/12, 17%), mental and behavioural disorders (2/12, 17%), visual field defect (1/12, 8%) and tremor (1/12, 8%), which were relatively unusual (Fig. 1). Six of 12 patients developed pneumonia (Table 2).

**Table 2:** Detailed characteristics of the 12 patients and the association of COVID-19 with neurological diseases

No. of patient	Age (years), gender	COVID-19 vaccination	Onset of pneumonia	Comorbidities	Initial neurological symptoms	CSF tests (1. pressure, cell count, protein level; 2. other tests)^a^	1. Autoimmune encephalitis antibody; 2. CNS demyelinating diseases antibody	EEG	MRI	Neurological diagnosis	Treatment (1. antiviral; 2. immunotherapy)	Outcome	The association of COVID-19 with neurological disease
1	31, F	Yes	No	N/A	Sensory or movement disorders	1. Normal 2. No OCB, SARS-CoV-2 PCR negative	1. Negative in serum and CSF 2. Negative in serum and CSF	N/A	Lesion in the right brachium pontis (Fig. 2)	Brainstem encephalitis	1. Acyclovir 2. IVMP (80 mg/day for 5 days) followed by CS taper	Initial improved then worsen, and improved after re-hospitalization	Brain stem encephalitis probably associated with SARS-CoV-2
2	37, M	Yes	Yes	N/A	Visual field defects	1. 170 mmH_2_O, 38 × 10^6^ /L, monocytes account for 99%, protein 852 mg/L 2. No OCB	1. Negative in serum and CSF 2. MOG-ab positive in serum	N/A	Left optic nerve was thickened	MOGAD	1. NO 2. IVMP (80 mg/day for 5 days, then 1 g/day for 5 days) followed by CS taper	Improved after low-dose CS therapy then worsen after high-dose CS therapy, and improved finally after CS taper	MOG-antibody-associated optic neuritis probably associated with SARS-CoV-2 infection
3	42, M	Yes	No	Hyperthyroidism	Memory deficits	1. Normal 2. No OCB	1. Negative in serum and CSF 2. Negative in serum and CSF	increased slow wave	No responsible cerebral lesion	Encephalitis	N/A	The follow-up revealed complete recovery	Probable SARS-CoV-2 encephalitis
4	23, M	Yes	No	N/A	Sensory or movement disorders	1. 230 mmH_2_O, 3 × 10^6^ cells/L, protein 650 mg/L 2. OCB positive in CSF and serum, elevated CSF IgG index (0.91) and 24-h IgG synthesis rate, SARS-CoV-2 PCR negative	1. Negative in serum and CSF 2. Negative in serum and CSF	N/A	Multiple cerebral lesions; no lesions in spinal cord (Fig. 2)	MS	1.NO 2.IVMP (500 mg/day for 5 days) followed by CS taper, IVIG 0.4 g/kg/day for 3 days	Improved gradually to baseline	MS probably associated with SARS-CoV-2 infection
5	15, F	Yes	No	N/A	Sensory or movement disorders	1. Normal 2. No OCB, elevated CSF IgG index (0.77)	1. N/A 2. MOG-ab positive in serum and CSF	N/A	Multiple lesions in periventricular white matter and spinal cord (Fig. 3)	ADEM	1. NO 2. IVMP (200 mg/day for 6 days) followed by CS taper	Complete recovery	ADEM probably associated with SARS-CoV-2 infection
6	35, F	No	Yes	Tumour	Seizure	N/A	N/A	Increased slow wave	Multiple reversible cortico-subcortical lesions (Fig. 4)	PRLS	1. NO 2. IVDM (10 mg/day for 15 days)	Complete recovered initially, while died of other complications during follow-up	Probable SARS-CoV-2 encephalitis
7	87, M	Yes	No	Encephalatrophy	Memory deficits	N/A	N/A	N/A	No responsible cerebral lesion	Encephalitis	1. Azvudine 2. Oral CS for 13 days	Improved gradually to baseline	Probable SARS-CoV-2 encephalitis
8	73, M	Yes	Yes	Hypertension	Memory deficits	N/A	1. Negative in serum 2. Negative in serum	Increased slow wave, suspicious three-phase wave (Fig. 5)	Extensive cortical lesions (Fig. 5)	Encephalitis	1.Azvudine 2. IVDM (5 mg/day for 3 days), then IVMP (80 mg/day for 9 days) followed by CS taper	Slight improvement with significant cognitive impairment remained	Probable SARS-CoV-2 encephalitis
9	73, F	Yes	Yes	Hypertension, diabetes, cerebral infarction	Seizure	1. 185 mmH_2_O, 27 × 10^6^/L, multinuclear cell count for 89%, protein 948 mg/L 2. N/A	1. Negative in serum and CSF 2. N/A	N/A	No responsible cerebral lesion	Encephalitis	1. Paxlovid and acyclovir 2. IVMP (80 mg/day for 5 days) followed by CS taper	Complete recovery	Probable SARS-CoV-2 encephalitis
10	51, M	Yes	Yes	Diabetes	Mental and behavioural disorders	N/A	N/A	N/A	No responsible cerebral lesion	Encephalitis	1. Azvudine and acyclovir 2. IVMP (60 mg/day for 6 days) followed by CS taper	Complete recovery	Probable SARS-CoV-2 encephalitis
11	51, F	Yes	No	Meningitis	Mental and behavioural disorders	1. 50 mmH_2_O, cell count and protein were normal 2. N/A	N/A	N/A	No responsible lesion	Encephalitis	1. Acyclovir 2. IVMP (80 mg/day for 6 days) followed by CS taper	Complete recovery	Probable SARS-CoV-2 encephalitis
12	62, M	Yes	Yes	N/A	Tremor	1. Normal 2. No OCB	1. Negative in serum and CSF 2. Negative in serum and CSF	Increased slow wave	No responsible lesion	Tremor	1. No 2. IVDM (5 mg/day for 4 days) followed by CS taper	Poor recovery	Tremor probably associated with SARS-CoV-2 infection

*M* male, *F* female, *N/A* not applicable, *CS* corticosteroids, *IVMP* intravenous methylprednisolone, *IVDM* intravenous dexamethasone

^a^CSF reference values: pressure: 80–180 mmH_2_O, cell count: 0–8 × 10^6^ /L, protein level: 120–600 mg/L, OCB: negative

**Fig. 2:**
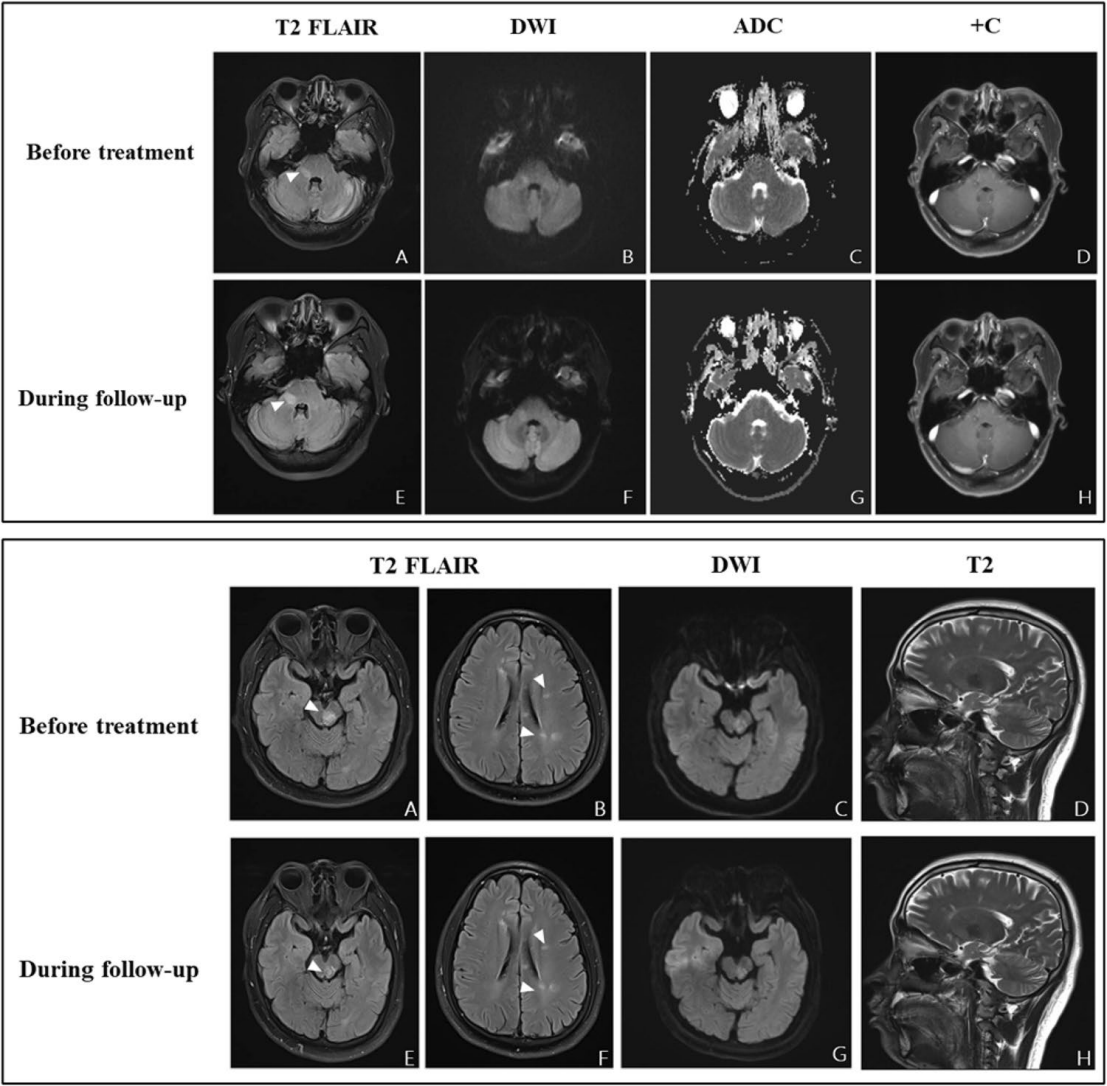
Up panel: Brain MRI of Patient 1. **A–D** show brain imaging examined before treatment, and **E**–**H** show imaging during follow-up period. **A**, **E** Hyperintensity in the right brachium pontis on T2-FLAIR; slightly increased intensity of the lesion on DWI (**B**, **F**) and ADC imaging (**C**, **G**). Punctate gadolinium enhancement of the lesion was observed on enhanced scan (**D**, **H**). Down panel: Brain MRI of Patient 4 **A**–**D** show brain imaging examined before treatment, and **E**–**H** show imaging during follow-up period. **A** Hyperintense lesion in the midbrain on T2-FLAIR and the signal was reduced during the follow-up (**E**); **C**, **G** The lesion presented hyperintense signal on DWI (**A**, **E**); **B**, **F** Hyperintense lesions distributed along the ventricle in axial imaging and sagittal imaging on T2W (**D**, **H**)

**Fig. 3:**
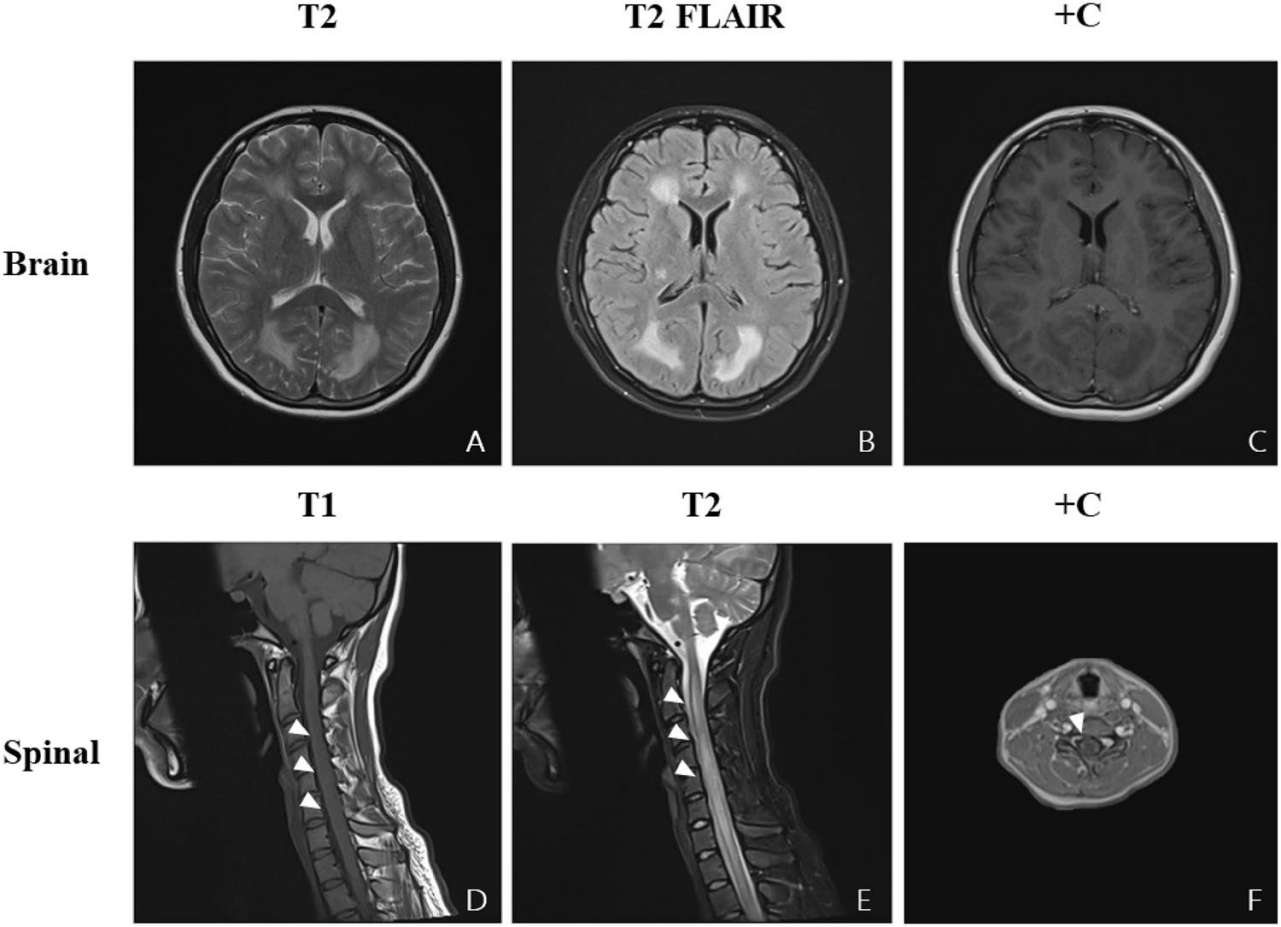
Brain and spinal cord MRI in Patient 5. **A**–**C** show the patient’s brain imaging, and **D**–**E** show spinal imaging. **A**, **B** Hyperintense signal presented in the periventricular white matter on T2 and T2-FLAIR; **C** no gadolinium enhancement was observed on the brain enhanced scan; **D** hypointense signal presented along the spinal cord on T1 and hyperintense signal presented in the same lesions on T2 (**E**); **F** circular hyperintense signal was found along the edge of spinal cord on enhanced scan

**Fig. 4:**
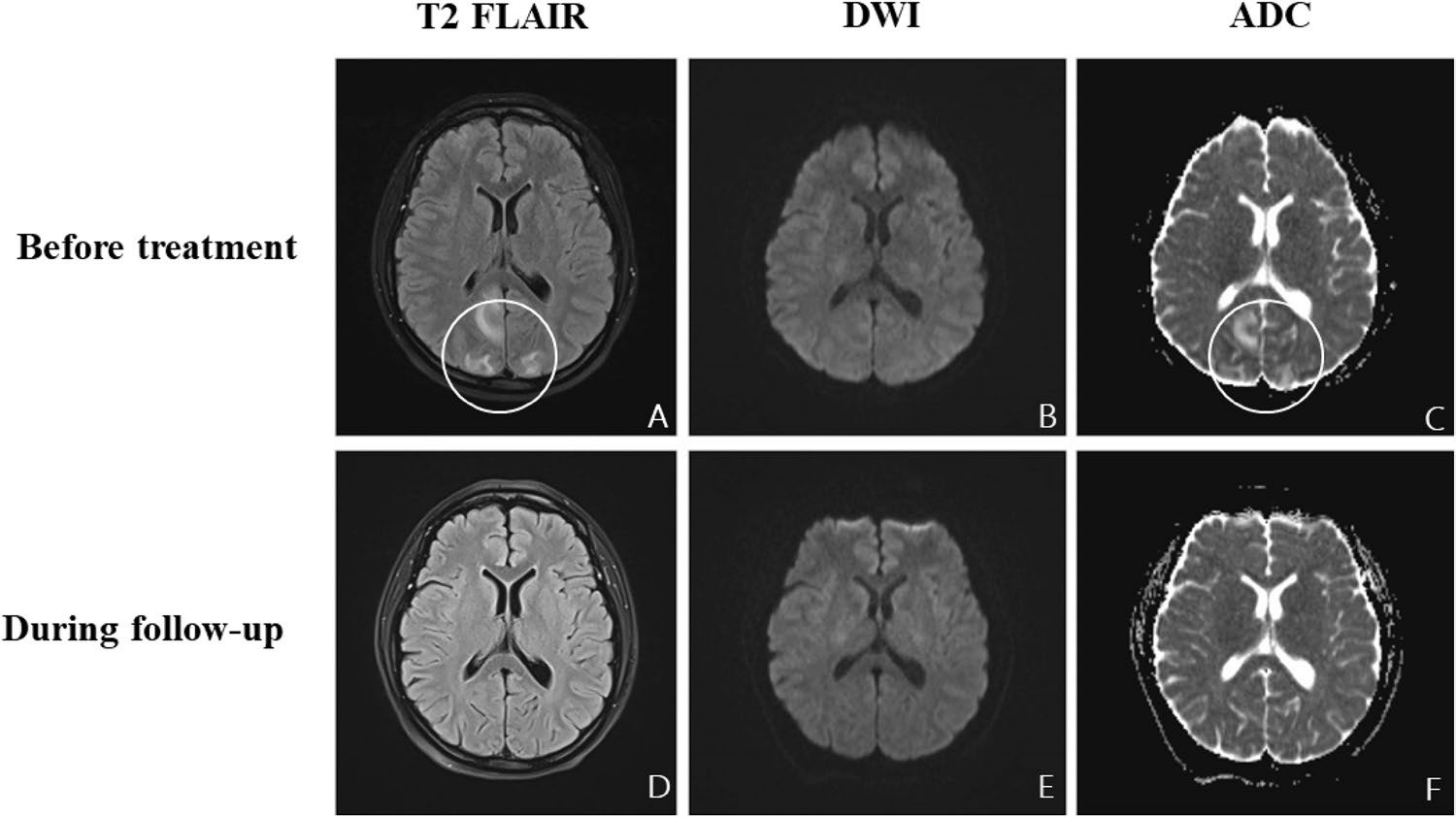
Brain MRI in Patient 6. **A–C** show brain imaging examined before treatment, and **D–F** show imaging during the follow-up period. **A** Hyperintensity present on bilateral parietal occipital lobes on T2-FLAIR and ADC imaging (**C**). No abnormal intensity was observed on DWI (**B**). **D–F** The lesions were fully absorbed, and no abnormal intensity was found on imaging taken during the follow-up period

**Fig. 5:**
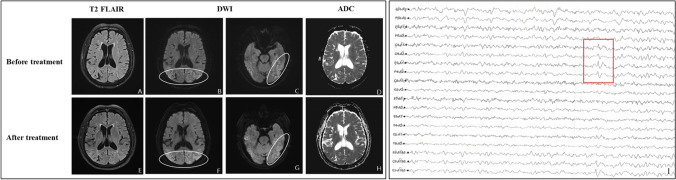
Brain MRI and EEG in Patient 8. **A–D** show brain imaging examined before treatment, and **E–H** show imaging during the follow-up period. **B**, **C**, **F**, **G** Hyperintense signal presented along the bilateral parietal occipital lobe cortex on DWI; **D**, **H** hypointensity presented in the same lesions as **B**, **F** on ADC imaging. **A**, **E** The lesions presented slightly hyperintense signal. **E**, **F** The lesions after treatment were more extensive than those before treatment (**A**, **B**). **I** EEG showed an increase in slow waves diffusely, and suspicious three-phase waves (red rectangle) were observed in the bilateral parietal, central and right occipital regions

All patients had laboratory evidence of SARS-CoV-2 infection. SARS-CoV-2 RT‒PCR tests on nasal swabs or throat swabs were positive in eight patients on admission, and three patients tested positive for SARS-CoV-2 RT‒PCR tests before admission. In addition, 1 patient who had not undergone SARS-CoV-2 RT‒PCR examination tested positive on a throat-swab antigen test before admission. All patients tested positive for serum SARS-CoV-2 IgG after admission.

All 12 cases underwent MRI scan. Six patients had abnormal MRI findings compatible with their diagnosis, and the other six patients had no responsible neuroimaging lesion. EEG examination was performed in four patients, all of whom showed an increase in background slow waves; one also had suspicious three-phase waves. Eight patients underwent lumbar puncture for CSF analysis; among them, three patients had elevated total white cell counts or protein quantifications with or without abnormal CSF pressure, one patient presented with low CSF pressure simply, and the remaining four patients had no obvious abnormalities. SARS-CoV-2 RT‒PCR was performed on the CSF of two patients (Patient 1, Patient 4), and the results were negative. Finally, eight patients were tested for autoimmune encephalitis antibodies or CNS demyelinating antibodies; serum myelin oligodendrocyte glycoprotein (MOG) antibody was positive in two patients and negative in six patients.

### Treatment and outcome

Of the eight patients (Patients 5–12) with positive SARS-CoV-2 RT‒PCR on admission, five patients (Patients 7–11) received antiviral therapy, while the remaining three patients (Patients 5–6, 12) did not receive antiviral therapy. Of the four patients (Patients 1–4) with negative RT‒PCR results on admission, only Patient 1 received antiviral therapy (Tables 1, 2). Paxlovid combined with acyclovir was used in Patient 9, Azvudine combined with ayclovir was adopted in Patient 10, Azvudine was used in Patients 7 and 8, and acyclovir was used in Patients 1 and 11. Eleven of the 12 patients received different doses of glucocorticoid therapy, of which 3 patients (Patients 4, 7, 9) received different doses of human immunoglobulin synchronously (Patient 4 was given an intravenous drip at a dose of 0. 4 g/kg/day for 3 days). Patient 3 was given medication for the underlying disease and received supportive treatment without any antivirals or immunotherapy.

All 12 patients were discharged from the hospital after their symptoms improved to different degrees. Of the 11 patients who received glucocorticoid therapy during hospitalization, 9 patients were treated with oral hormones that were tapered off after discharge. At the first follow-up visit (1 month after discharge), nine patients recovered well, while the other three patients (Patients 3, 8, 12) showed unsatisfactory improvement in clinical symptoms with remaining significant neurological impairment. At the second follow-up visit (2 months after discharge), eight patients recovered well without obvious sequelae, two patients (Patients 8, 12) had residual neurological dysfunction, and one patient (Patient 1) relapsed during glucocorticoid reduction and improved again after receiving rehospitalization for glucocorticoid therapy. Unfortunately, the patient with a rectal tumour passed away due to multiple complications associated with the tumour.

The clinical characteristics of these patients are summarized in Table 2 in detail.

## Discussion

This is an observation study of novel coronavirus-related central nervous system complications, including data from 12 patients admitted to the inpatient department of Neurology, the Eighth Affiliated Hospital, Sun Yat-Sen University during the epidemic period. The study summarized and analysed the clinical manifestations, laboratory and imaging examination results, treatment and outcomes of 12 patients, providing a reference for future medical treatment of CNS complications related to the novel coronavirus in the neurology department in order to achieve early disease identification, early diagnosis, systematic evaluation and treatment, and ultimately obtain a better prognosis. The overarching goal is to minimize the negative long-term impact of COVID-19.

There are seven coronaviruses currently known to cause disease in humans, and four of them—229E, OC43, NL63 and HKU1—usually cause mild seasonal respiratory symptoms in humans [20, 21]. The remaining three coronaviruses can lead to a severe acute respiratory disease pandemic: SARS-CoV, which caused the SARS outbreak [22]; MERS-CoV, which caused Middle East respiratory syndrome (MERS) [23]; and SARS-CoV-2, which is currently causing COVID-19. In contrast to the six other coronavirus subtypes, SARS-CoV-2 is classified as a β-coronavirus. SARS-CoV-2 shares 79.5% of its sequence with SARS-CoV [24], but its affinity for angiotensin-converting enzyme-2 (ACE2) is 10–20 times higher than that of SARS [25]. ACE2 is widely distributed in vascular endothelial cells and arterial smooth muscle cells of human organs [26]. In 2020, Rongrong Chen et al. found that ACE2 is expressed in many neurons and some non-neuronal cells (mainly astrocytes, oligodendrocytes, and neurovascular endothelial cells) of the brain [27], which makes the CNS a target organ for SARS-CoV-2. According to prior research, SARS-CoV-2 infection can activate the innate immune response uncontrollably while impairing adaptive immunity [28]. The excessive systemic inflammatory response caused by innate immune maladaptation can damage the function of the CNS by impairing the function of neurovascular endothelial cells, activating the CNS innate immune signalling pathway, and inducing autoimmunity [29]. All these features may contribute to a relatively high clinical prevalence of COVID-19-associated CNS complications.

A wide variety of neurological syndromes have been reported in COVID-19 cases, including encephalitis [9, 30–33], encephalopathy, stroke, ADEM [14], reversible posterior leukoencephalopathy [34–36], optic neuritis [37, 38], and epilepsy, among other. With reference to the standard case definitions for the association of COVID-19 with neurological disease [39], 7 of 12 patients (Patient 3, Patients 6–11) can be categorized as having probable SARS-CoV-2 encephalitis, 1 patient (Patient 1) can be described as having brain stem encephalitis probably associated with SARS-CoV-2 infection, 1 patient (Patient 5) can be described as having ADEM probably associated with SARS-CoV-2 infection, 1 patient (Patient 2) can be described as having MOG-antibody-associated optic neuritis probably associated with SARS-CoV-2 infection, 1 patient (Patient 4) can be described as having MS probably associated with SARS-CoV-2 infection, and the remaining patient (Patient 12) can be described as having tremor probably associated with SARS-CoV-2 infection.

### Encephalitis and brainstem encephalitis

#### Encephalitis

Encephalitis is inflammation of the brain parenchyma with clinical evidence of neurologic dysfunction characterized by altered mental states, fever, seizures, focal neurologic defects, proliferation of CSF white blood cells, and abnormalities in neuroimaging and EEG [40, 41]. A study of 203 patients with encephalitis showed that 42% had infectious causes, 21% were immune mediated, and the remaining had unknown causes [42].

The proposed neurotropic mechanisms of COVID-19 leading to neuropathology can be mainly summarized as direct damage caused by viral infection [43–45] and indirect damage mediated by inflammation or autoimmunity [19, 28]. A systematic review [11] generalized that the most frequent neurological manifestations of encephalitis in patients with COVID-19 were seizure (29.5%), confusion (23.2%), headache (20.5%), disorientation (15.2%), and altered mental status (11.6%). Approximately 60–70% of patients have abnormal findings in MRI, EEG, and CSF [11]. EEG is mostly nonspecific, with slow background activity [11]. The changes in MR imaging varied greatly based on different cases. It is difficult to isolate the virus from CSF [11]. In our study, the relatively common symptoms in these seven patients with encephalitis were cognition impairments (5/7, memory deficits and unresponsiveness), mental and behavioral disorders (4/7), disorders of consciousness (2/7) and seizures (2/7). Different forms of lesions were observed in five patients, and no responsible lesions were observed in the other two patients. Two of the five patients who underwent lumbar puncture showed abnormal CSF pressure, and one patient also showed elevated levels of white blood cells and proteins. Increased slow waves were observed in all three patients who had undergone EEG examination, and suspicious three-phase waves were observed in one of them. The symptoms were almost completely relieved in five of the seven patients at the follow-up 2 months after discharge, while the symptoms of Patient 7 were slightly relieved with significant cognitive dysfunction remaining. In addition, the patient who was diagnosed with reversible posterior leukoencephalopathy with tumour had no neurological symptoms at the first follow-up visit (1 month after leaving our Department of Neurology), while she had passed away by the time of the second follow-up visit.

#### Brainstem encephalitis

Patients with brainstem encephalitis can have no consciousness dysfunction or mental disorder and thus might not strictly fulfill the proposed diagnostic criteria for encephalitis [40, 46]. Brain stem encephalitis has been reported in COVID-19 patients [47]. The aetiological mechanism of brainstem encephalitis has not been elucidated, and most scholars believe that it is associated with infection. The most common clinical symptoms were cranial nerve damage and pyramidal tract signs. Brain MRI can show lesions in the brain stem, among which the pontine is the most common site, with a high FLAIR signal and equal or slightly high signal on DWI. The lesion can be gadolinium enhanced or not. Glucocorticoid therapy is effective [48, 49]. Our study reported a patient with brain stem encephalitis probably associated with SARS-CoV-2 infection, of whom the neurological symptoms developed 20 days following the presence of respiratory symptoms. The main clinical manifestations were trigeminal nerve and vestibular dysfunction. The lesions in the right brachium pontis presented high signals in both FLAIR/T2W and DWI with spot-like and line-like gadolinium enhancement in the enhanced scan. CSF analysis was completely normal. This patient responded well to glucocorticoid therapy.

### Idiopathic demyelinating diseases

In our study, Patient 5 can be described as having ADEM probably associated with SARS-CoV-2 infection, Patient 2 can be described as having MOG-antibody-associated optic neuritis probably associated with SARS-CoV-2 infection, Patient 4 can be described as having MS probably associated with SARS-CoV-2 infection. All three patients were classified as idiopathic demyelinating diseases of the CNS, and two of them had MOG-antibody-associated disease (MOGAD). Idiopathic demyelinating disease of the CNS is a group of CNS demyelinating diseases characterized by pathological manifestations of demyelinating and inflammatory cell infiltration, including MS, neuromyelitis spectrum disease, ADEM, and other different clinical phenotypes [50]. Viral infections are considered a possible triggering factor [51]. Several case reports of demyelination of the CNS in COVID-19 patients have been published [14, 37, 38, 52–54]. A systematic review showed that the median duration between COVID-19 infection and demyelinating event onset was 6 days (range − 7 to + 45 days) in MOGAD and 13.5 days (range − 21 to + 180 days) in MS [51]. In our study, the delay before the onset of neurological symptoms measured 12 days and 35 days in the 2 patients of MOGAD and 25 days in MS (Fig. 1). It was reported that the most common presenting neurological symptoms were lethargy and sensory alterations with or without seizures [55]. Among our three patients, two patients mainly manifested as sensor or motor disorders and the other patient presented visual field defects.

#### MOGAD

Myelin oligodendrocyte glycoprotein (MOG) is a minor protein localized at the outermost layer of the myelin sheath and oligodendrocyte [56]. Pathogenic MOG-Abs can be present in the circulation and have no effect unless they gain access to the CNS via an opening of the blood–brain barrier (BBB) that results from an inflammatory environment [57]. Diffuse microvessel endothelial damage in the brain has been described in human COVID-19 cases [58]. Evidence for altered BBB permeability following SARS-CoV-2 infection has been obtained in in vitro BBB models [59] and in vivo animal models [58, 60]. Markers of BBB dysfunction such as SUR1 and TRPM4, were increased in endothelial cells in COVID-19 brains [60]. A hypothesis was proposed that hyperinflammation provoked by maladaptive innate immunity may impair neurovascular endothelial function, disrupt the BBB, activate CNS innate immune signalling pathways, and induce para-infectious autoimmunity, potentially contributing to the CNS complications associated with SARS-CoV-2 infection [29]. The most common clinical phenotype of MOGAD is ADEM in young children and optic neuritis in children aged > 9 years and adults [57]. Oligoclonal bands (OCBs) in the CSF were usually negative in patients who were MOG Ab + [61]. Most patients with MOGAD recover well from attacks [57]. In our two MOGAD patients, one was an adolescent female presenting with ADEM, and the other was an adult male presenting with ON. CSF oligoclonal zone analysis presented abnormal local immune globulin synthesis with BBB dysfunction in both patients, and the oligoclonal band was negative in both blood and CSF. Both of them recovered well after treatment with glucocorticoids.

#### Multiple sclerosis

Multiple sclerosis (MS) is a chronic inflammatory demyelinating disease of the CNS that gives rise to focal lesions in the grey and white matter and to diffuse neurodegeneration in the entire brain [62–64]. BBB disruption and microglial activation were observed in perivenous MS lesions [65]. The altered BBB permeability following SARS-CoV-2 infection and microglial apoptosis detected in a mouse model of COVID-19 suggest that the occurrence of MS in COVID-19 patients is not a mere coincidence. There have been several case reports of MS following SARS-CoV-2 infection [53, 66–68], and cytokines involved in MS are upregulated in COVID-19 patients. In our study, brain MRI of this MS patient showed bilateral lateral ventricle, corona radiata and midbrain lesions, none of which were enhanced with gadolinium. OCBs were positive in both blood and CSF, and there was more OCB in CSF than in blood. In addition, the IG synthesis index and the 24 h synthesis rate of CSF were elevated. Both the AQP4 and MOG antibodies were negative. These findings all support the diagnosis of MS.

### Tremor

In our study, the main neurological manifestation of Patient 12 was tremor, and the follow-up visits showed unsatisfactory improvement of symptoms. Tremor has been reported in patient with COVID-19 [69], and hyposmia is the early symptom of SARS-CoV-2 infection [70]. Notably, tremor is a main clinical manifestation of Parkinson's disease (PD), and hyposmia is a preclinical symptom of PD [71]. In addition, the host-cell receptor of SARS-CoV-2—ACE2—is highly expressed in the substantia nigra [27], which is the location of characteristic pathological changes in PD. All this information reminds us to think carefully about whether SARS-CoV-2 can cause tremors or accelerate the progression of PD carefully. However, there is no direct evidence to clarify the association between SARS-CoV-2 infection and tremor or PD [72], and further long-term studies are needed.

This study has some limitations. First, as our study unit was the inpatient department of neurology, the number of patients included was relatively insufficient, which precluded detailed analysis and summary of the clinical characteristics of each neurological complication. Furthermore, the aetiopathological role of SARS-CoV-2 in the observed CNS complications cannot be illustrated.

However, the causality of the association between SARS‐CoV‐2 infection and the development of these CNS complications cannot be made with absolute certainty. In the setting of the SARS‐CoV‐2 pandemic, we cannot consider CNS complications in COCID-19 patients as coincidence by mistake. In this fight against the pandemic, we need up-to-date systematic reviews and scientific research to constantly arm health workers.

## Data Availability

The data are available from the corresponding author on reasonable request.
